# Determinants of Consumers’ Behavior in Reducing Pesticide Residues in Vegetables and Fruits, Northern Thailand

**DOI:** 10.3390/ijerph192013033

**Published:** 2022-10-11

**Authors:** Ratana Sapbamrer, Jiraporn Chittrakul

**Affiliations:** Department of Community Medicine, Faculty of Medicine, Chiang Mai University, 110 Inthavaroros Road, Sri Phum Subdistrict, Mueang District, Chiang Mai 50200, Thailand

**Keywords:** pesticide, vegetable, fruit, consumer, food safety, behavior, rural, urban

## Abstract

Pesticide residues in vegetables in northern Thailand exceed the maximum residue limits established by the European Union. Therefore, improved knowledge and behavior in reducing pesticide residues in vegetables and fruits (VF) would reduce the risk of exposure to pesticides. This study aims to investigate the contributing factors of consumers’ behavior in reducing pesticide residues in VF. The differences in knowledge, attitude, and behavior in reducing pesticide residues in VF between consumers living in rural and urban communities of Chiang Mai, Thailand were also investigated. The cross-sectional study was carried out during August and October 2021 with 456 participants. Data was collected from participants using a Google form questionnaire. The results indicated that pesticide-free was the top-ranked consideration for VF purchasing. Linear regression analysis found that factors associated with consumers’ behavior in reducing pesticide residues in VF were total knowledge scores (Beta (β) ± standard error (SE.) = 1.15 ± 0.18, 95%CI = 0.79, 1.51), total attitude scores (β ± SE. = 1.30 ± 0.49, 95%CI = 3.87, 10.40), having co-morbidity (β ± SE. = 3.2 ± 1.37, 95%CI = 0.52, 5.90), type of VF purchasing (β ± SE. = 1.98 ± 0.57, 95%CI = 0.85, 3.11), frequency of VF purchasing (β ± SE. = 3.81 ± 1.18, 95%CI = 1.49, 6.13), price of VF products (β ± SE. = −2.23 ± 1.13, 95%CI = −4.45, −0.02), and getting information from the broadcasting tower in the village (β ± SE. = 7.13 ± 1.66, 95%CI = 0.32, 2.27).

## 1. Introduction

The increase in the world’s population in the 20th century is the major cause of the increased demand in food production. Meanwhile, the agricultural use of pesticides has increased significantly to control pests and increase crop yields worldwide, to ensure there is enough food for the world’s population [1]. However, pesticides have adverse effects on environments, ecosystems, and humans. Exposure to pesticides can cause acute and chronic health effects, depending on doses of exposure, types of pesticides, and route of entry, including cancers, diabetes, chronic kidney disease, asthma, and neurological diseases [2,3,4,5,6]. Ingestion is the main route of exposure in the general population and consumers, who are primarily exposed to pesticides through eating food and drinking water contaminated with pesticide residues [3,4,5,6,7].

The National Bureau of Agricultural Commodity and Food Standards, Ministry of Agriculture and Cooperatives issued a revised announcement entitled: “Thai agricultural standards, pesticide residues: Maximum Residue Limits (MRLs) (TAS 9002-2016)” in 2016 [8]. However, because these notices are updated every five to ten years, they are usually out of date in terms of safeguarding consumer health [9]. Due to traders’ illicit stock, high demand from farmers, and smuggling across the Thai border, illegal pesticide use in agriculture is still a problem [10]. A previous study investigated organophosphate residues in vegetables in northern Thailand and found that 59.3% of vegetables from farms and 13.2% from local markets had organophosphate residues exceeding the MRL established by the European Union [11]. Wanwimolruk et al. [12] also investigated organophosphate and carbamate residues in vegetables in central Thailand and found that 42–71% of vegetables from local markets and 33–55% from supermarkets had pesticide residues exceeding the EU MRL. In addition, a study by Liu et al. [13] suggested that the consumption of fruits and grains was associated with increased levels of urinary organophosphate metabolites in urban pregnant women.

Previous studies regarding the factors influencing consumer choice in vegetable and fruit (VF) purchases suggested that price, freshness, and appearance were the major factors [14,15]. Consumer income was also a factor influencing VF purchasing behavior. Consumers with higher incomes preferred to purchase off-season, processed, pesticide-free, organic vegetables from supermarkets, due to health concerns. However, organic labels and the brand of products appeared to be unimportant in VF purchasing decisions. The presence of children in a family influenced consumers’ behavior in selecting safe VF [15]. Consumers were willing to pay a premium price for selecting safe food [16,17]. Consumers who had high levels of food safety knowledge could improve their attitude and practice towards food purchasing in shops and in cooking [18]. Increasing knowledge through these different channels could have a beneficial impact on changing a consumer’s understanding of health, resulting in modifying consumer behavior [19]. In addition, the living region of consumers was also the principal determinant of food safety concerns [20] Although most available studies investigate factors influencing VF purchasing, the studies regarding factors influencing behavior in reducing pesticide exposure from VF consumption remain scarce [14,15]. Therefore, improved knowledge and behavior regarding methods used by consumers for choosing VF and reducing pesticide residues in VF may reduce the risk of exposure. The theoretical framework is presented in Figure S1.

Chiang Mai Province is in the northern part of Thailand, 720 km far from Bangkok (capital city of Thailand), and covers an area of 20,170 sq.km. Chiang Mai Province is the largest and capital city of northern Thailand. Therefore, this Province is a central part of the business, logistics, and tourism of northern Thailand [21]. This study aims to investigate the contributing factors of consumers’ behavior in reducing pesticide residues in VF. The differences in knowledge, attitude, and behavior in reducing pesticide residues in VF between consumers living in rural and urban communities of Chiang Mai, Thailand, were also investigated. The findings are useful for designing appropriate health-promoting activity on a basis of characteristics of consumers.

## 2. Materials and Methods

### 2.1. Study Design and Participants

This study was a cross-sectional investigation made up of people who live in the Chiang Mai Province of northern Thailand. An online survey (a Google form questionnaire) was distributed to all districts in Chiang Mai through online social media platforms. Inclusion criteria were as follows: (1) lived in one of the twenty-five districts of the Chiang Mai Province; (2) aged 18 years or older; and (3) bought and/or eat VF. Individuals illiterate in the Thai language were excluded. A sample size was estimated using the Taro Yamane formula with an alpha level (α) of 0.05 and a margin of error of 0.05.

The formula of Taro Yamane is as follows [22]:

n = N/(1 + Ne^2^)

where:

n = the sample size
N = the population size
e = the acceptable sampling error

The calculated recommended sample size was at least 400. A convenience sample was conducted during August and October 2021, and 456 consumers responded. Mueang Chiang Mai District of Chiang Mai Province is a central part of the business, logistics, and tourism industries of the Chiang Mai Province. Therefore, respondents from this district were classified as urban consumers. Respondents in other 24 districts, including Mae Ai, Fang, Chai Prakan, Wing Haeng, Chiang Dao, Phroa, Mae Taeng, Doi Saket, San Sai, Mae Rim, Samoeng, Galyani Vadhana, Hang Dong, Mae On, Saraphi, San Kampaeng, San Pa Tong, Mae Wang, Doi Lo, Mae Chaem, Chom Thong, Hot, Doi Tao, and Omkoi Districts, were classified as rural consumers. The 456 respondents covered all districts of the Chiang Mai Province (Figure S2).

### 2.2. Questionnaire

Data was collected from participants using a Google form questionnaire. The questionnaire took 10–15 min to complete and participants completed it themselves. The questions in the questionnaire were created according to the theoretical framework and previous studies [14,15,16,17,18,19,20]. The form of the questionnaire is presented in Supplementary Materials. This questionnaire was composed of five parts, including:

(1) Socio-demographic characteristics: age, gender, marital status, education, occupation, monthly income, co-morbidity, living area, and smoking and alcohol drinking habits.
(2) Habits of VF consumption. There were 5 questions. The questions were asked about type, frequency, and source of VF purchasing, source of information about pesticides, and considerations for purchasing VF.
(3) Knowledge about reducing pesticide residues in VF. There were 21 questions. The questions asked about knowledge regarding types of VF products, purchasing VF products, cleaning of VF products, and health effects from pesticide exposure.
(4) Attitude towards pesticide residues in VF. There were 6 questions. The questions asked about the respondent’s attitude towards health effects, and environmental effects from pesticides. The questions were from a focus group discussion, and were designed using a dichotomous scale (yes/no).
(5) Behavior in reducing pesticide residues in VF. There were 33 questions. The questions were about self-reported behavior and designed using Likert rating scales with scores from 1 to 4, and asked about types of VF products, purchasing VF products, cleaning of VF products, and cooking methods of VF products.

The questionnaire tested the validity and reliability of the questions before collecting the data. The index of congruence (IOC) score for each question of the questionnaire was higher than 0.5. The total reliability coefficient for all the questions was 0.893.

### 2.3. Statistical Analysis

Descriptive statistics, including frequency (n), percentage (%), mean and standard deviation (SD.), median, 25th percentile (P^25th^), and 75th percentile (P^75th^) were used to present demographic characteristics, knowledge, attitude, and behavior in reducing pesticide residues in VF. Normal distribution of data was tested before analyzing inferential statistics. An independent *t*-test was used for normal distribution of data (such as age, knowledge of types and cleaning of VF products, behavior regarding types, purchasing, and cleaning of VF products). A Mann–Whitney U test was used for non-normal distribution of data (such as knowledge regarding purchasing of VF products, knowledge of health effects from pesticides, total attitudes, behavior regarding method of cooking). Chi-square tests were used for categorical data. Multiple linear regression using a stepwise method was used to study the factors associated with behavior in reducing pesticide residues in VF. All determinant variables were tested for their association with the behavior before including the variables in the model. The variables which had the most significant association with the behavior (*p* value < 0.05) were incorporated into the model. Beta (β) and standard error (SE.) were presented. The Jamovi version 16.6 program was used to analyze the data.

## 3. Results

### 3.1. Demographic Characteristics

Table 1 presents the demographic characteristics and habits of VF consumption among rural and urban consumers (*n* = 456). The results found that 71.5% of respondents were female, 58.1% were married, 79.4% had graduated with bachelor’s degree or higher, 46.5% had an income of 285–857 US Dollars per month, and 75% were officers. Almost all consumers (96.5%) did not smoke cigarettes, 75.4% did not drink alcohol, and 78.3% had no co-morbidity. A high proportion of rural consumers were married (62.4%) and had children in the family (57.1%), while a high proportion of urban consumers were found to have a bachelor’s degree or higher (89.7%) and a monthly income higher than 857 US dollars per month (51.5%).

### 3.2. Habits of VF Consumption

The results found that 61.6% of consumers sometimes purchased VF, 76.3% purchased VF from markets, and 40.4% purchased organic VF. When comparing the habits among rural and urban consumers, urban consumers purchased organic VF (53.6%) more frequently than rural consumers (36.8%). Rural consumers purchased VF from markets (80.2%) and planted VF themselves (6.4%) more frequently than urban consumers (61.8% and 2.1%, respectively) (Table 1).

**Figure 1:**
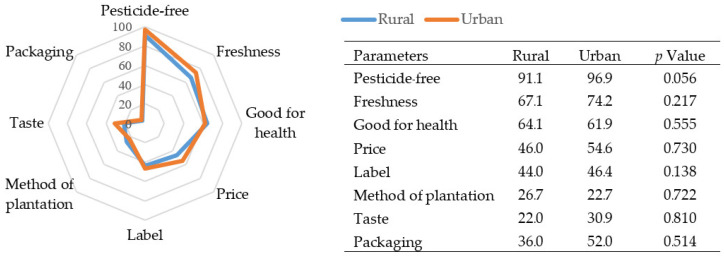
Percentage (%) of considerations for purchasing VF.

**Figure 2:**
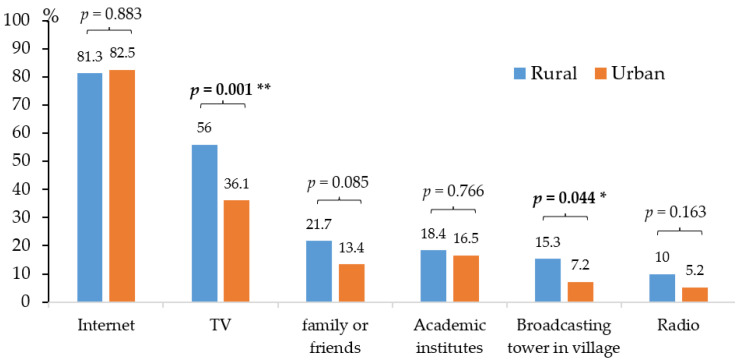
Source of information regarding reducing pesticide residues in VF, * *p* value < 0.05, ** *p* value < 0.01.

Figure 1 presents the list of considerations of consumers for VF purchasing. Most consumers chose to purchase pesticide-free VF (91.1% for rural and 96.9% for urban); the second highest consideration was freshness (67.1% for rural and 74.2% for urban), followed by health (64.1% for rural and 61.9% for urban, respectively). When asked about the source of their information about pesticides, most consumers got the information from the internet (81.3% for rural and 82.5% for urban), followed by TV (56% for rural and 36.1% for urban). Rural consumers got information from TV (56%) and from a broadcasting tower in their village (15.3%), a significantly higher level than urban consumers (36.1% and 7.2%, respectively) (Figure 2).

### 3.3. Knowledge, Attitude and Behavior in Reducing Pesticide Residues in VF

**Figure 3:**
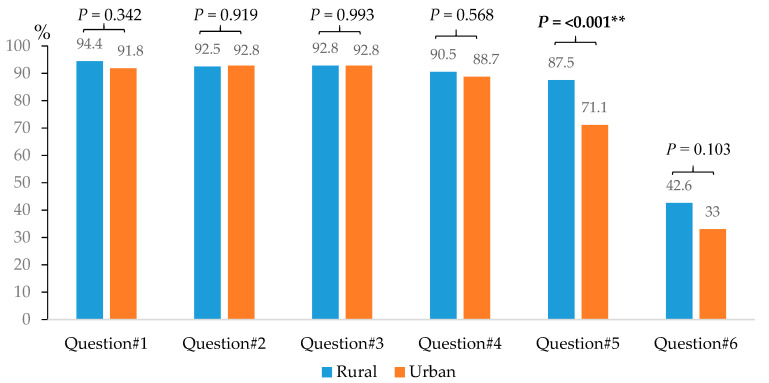
Attitude of consumers regarding pesticide residues in VF, ** *p* value < 0.01. Question #1. Do you worry about the effects of pesticide residues in VF on own health? Question #2. Do you worry about the effects of pesticide residues in VF on your children’s health? Question #3. Do you think that purchasing non-pesticide VF is worthwhile? Question #4. Do you worry about the effects of pesticide residues in VF on the environment? Question #5. Do you need to plant VF for your own consumption? Question #6. Do you feel sick because of pesticide residues in VF?

Rural consumers had significantly higher knowledge, attitude, and behavior scores than urban consumers when considering the types of VF products. Behavior scores regarding the cleaning of VF products and cooking methods in rural consumers were significantly higher than those in urban consumers (Table 2). The results also indicated that rural consumers needed to plant VF for their own eating significantly more often than urban consumers (87.5% for rural and 71.1% for urban) (Figure 3).

### 3.4. Determinants of Consumers’ Behavior in Reducing Pesticide Residues in VF

Multivariate analysis found that factors associated with consumers’ behavior in reducing pesticide residues in VF were total knowledge scores (β ± SE. = 1.15 ± 0.18, 95%CI = 0.79, 1.51), total attitude scores (β ± SE. = 1.30 ± 0.49, 95%CI = 3.87, 10.40), having co-morbidity (β ± SE. = 3.2 ± 1.37, 95%CI = 0.52, 5.90), type of VF purchasing (β ± SE. = 1.98 ± 0.57, 95%CI = 0.85, 3.11), frequency of VF purchasing (β ± SE. = 3.81 ± 1.18, 95%CI = 1.49, 6.13), price of VF products (β ± SE. = −2.23 ± 1.13, 95%CI = −4.45, −0.02), and getting information from the broadcasting tower in the village (β ± SE. = 7.13 ± 1.66, 95%CI = 0.32, 2.27) (Table 3).

## 4. Discussion

The main finding of this study is that factors associated with consumers’ behavior in reducing pesticide residues in VF were total knowledge scores, total attitude scores, having co-morbidity, type of VF purchasing, frequency of VF purchasing, price of VF products, and getting information from the broadcasting tower in the village. Additionally, VF being pesticide-free was ranked a top consideration of consumer purchasing. It is possible that consumers perceived the chemical hazards associated with pesticide use to have a negative impact on their health [20,23]. In addition, the results show that both rural and urban consumers were concerned about the effects of pesticides on their health, their children’s health, and the environment. The results also found that rural consumers needed to plant VF for their own eating significantly more than urban consumers. This may be due to rural consumers having more space and land available for living and planting compared with urban consumers. Rural consumers also had higher knowledge, attitude, and behavior scores regarding reducing pesticide residues in VF than urban consumers. A previous study by Ha et al. [20] suggested that the living region of consumers was the principal determinant of food safety concerns. They also identified that urban consumers had higher perception scores regarding food safety than their rural consumers. This contradiction may be explained by differences in consumer demographic characteristics. This study found that a higher proportion of rural consumers were married and had children compared with urban consumers. These results were similar to those identified by Massaglia et al. [15] which suggested that the presence of children in a family influenced consumers’ behavior to select safe VF. In addition, rural consumers usually lived in more cultivated areas, therefore were more frequently exposed to pesticides, and consequently may have been more aware of the adverse effects of pesticide exposure on their own and their family’s health.

This study also identified that the majority of rural consumers bought VF from markets, and chose pesticide-free VF, while the majority of urban consumers bought VF from supermarkets and chose organic VF. This suggests that urban consumers have a higher perception of safe food than rural consumers. In fact, some consumers did not clearly understand the definition of VF products. In Thailand, there are several names for VF products, including safe VF, healthy VF, pesticide-free VF, limited pesticides used, chemical VF, hydroponic VF, and organic VF. This variation appeared to confuse consumers. This makes it difficult for consumers to know whether VF products are safe [24]. Government certification and supplier indicators appear to play a vital role in building consumers’ trust [25,26]. Consistent certification would ultimately reduce production costs and be of benefit to the environment and consumer health [26]. Consumer education regarding VF definition and its labelling would also be of benefit. In addition, education and an awareness program should be developed, suited to living region characteristics of consumers [27]. For example, effective communication to urban consumers should address how to select, clean, and cook VF in a way that is safe for their health. Information regarding alternatives for cleaning VF, such as using alkaline solution, sodium bicarbonate, ozone, and other methods is also required [28,29,30].

The findings of this study were that the key factors associated with consumer behavior in reducing pesticide residues in VF were knowledge level, attitude level, having co-morbidity, type and frequency of VF purchasing, price of VF products, and source of information. Knowledge is distinctively relevant to attitude and behavior and is crucial for changes in attitude and behavior [19]. The complexity of knowledge and attitude affects a consumer’s decision to act [31]. The results of this study demonstrate that total knowledge and attitude scores were positively correlated with consumer behavior towards reducing pesticide residues in VF. These results agreed with the study by Mihalache et al. [18] which suggested that consumers who had high levels of food safety knowledge could improve their attitude and practice towards food purchasing in shops and in cooking. Increasing knowledge through these different communication channels could have a beneficial impact on changing a consumer’s understanding of health, resulting in modifying consumer behavior [19].

Co-morbidity was recorded as a crucial factor for consumer behavior. The results of this study indicated that co-morbidity was closely associated with consumer behavior in reducing pesticide residues in VF. These results aligned with previous studies which claimed that co-morbidity was directly associated with health intention and behavior [32,33]. These findings may be useful for promoting health behavior in this co-morbid population.

VF purchasing factors were determinants of consumers’ behavior towards reduction in pesticide residues in VF. The results of this study indicated that consumer behavior was positively associated with type and frequency of VF purchasing, but negatively associated with the price of VF products. These results implied that consumers who always purchased VF and purchased organic or pesticide-free VF were more likely to have high behavior scores than the ones who did not. These results were consistent with the study by Cheng et al. [34] which suggested that frequency of vegetable purchasing was associated with food safety concerns. In addition, previous studies revealed that consumers were willing to pay a premium price for selecting safe food [16,17]. A meta-analysis by Massey et al. [35] also suggested that consumers’ intention to purchase organic food is higher when they perceive organic food to be expensive. These findings gave useful information for agricultural production sectors in selecting planting strategies for safe VF production.

The source of information regarding reducing pesticide residues in VF was recorded as an important factor for consumers’ behavior. The results of this study found that most consumers in both rural and urban communities got their information from the internet. This implies that rural and urban consumers had the potential to access the internet to get the information. These results provided a useful insight into potentially effective channels to provide food safety information to consumers. Importantly, these results highlighted that consumers who got their information from a broadcasting tower in their village were more likely to have higher behavior scores than the ones who did not. In addition, rural consumers who got their information from a broadcasting tower had significantly higher behavior scores than urban consumers. Broadcasting towers in Thailand are used to communicate a range of information which includes health education information. This communication is usually used in rural areas and is administrated by a village head. A previous study suggested that the content of broadcasting, the participation of the community, and support from other organizations were crucial factors for the sustainable development of a broadcasting tower administration [36]. This study therefore suggested that the broadcasting tower in a village is an important channel to communicate food safety information, especially in rural areas.

The sample of this study covered all 25 districts of Chiang Mai Province; therefore, it is reasonable to suggest this sample could be extrapolated to represent the entire population of the Chiang Mai Province. However, some limitations need to be considered. Firstly, a Google form questionnaire was used for collecting the data from the study participants; as a result, the study was confined to literate persons with internet access. Secondly, a quantitative research design may not provide the insight data. Therefore, additional qualitative research maybe warranted to provide a more comprehensive conclusion. Thirdly, the target population in this study was selected only from VF consumers. However, a study using non-VF consumers as a control group might improve the efficiency of the existing information-conveying strategies to better engage their audiences. Therefore, a study with non-VF consumers requires more investigation in further research. Fourthly, the questions in the questionnaire were created according to the theoretical framework and previous studies, and the IOC score and reliability coefficient for each question in questionnaire were assessed before collecting the data. However, this study did not measure test–retest reliability. The test-retest reliability ensures that the measurements collected in one sitting are both representative and consistent across time. As a result, the test–retest reliability should warrant further investigation. Finally, self-reported behavior was employed in this study. Cognitive bias, egocentrism, and other empowering factors may impact the self-reporting of behavior [37,38]. Therefore, an observed behavior approach may be beneficial for collecting actual behavior, and warrants further investigation.

## 5. Conclusions

Produce being pesticide-free was a primary consideration for VF purchasing. Most consumers accessed their information about pesticides through the internet and were concerned about the effects of pesticides on their health. Rural consumers had significantly higher knowledge, attitude, and behavior scores than urban consumers. Importantly, factors associated with consumer behavior in reducing pesticide residues in VF were knowledge, attitude, having co-morbidity, type and frequency of VF purchasing, price of VF products, and source of information. Understanding the factors influencing consumers’ behavior in reducing pesticide residues in VF will develop suitable targeted intervention strategies for education and awareness-raising campaigns focusing on selecting types of VF products, cleaning methods for VF, and health effects from pesticide exposure. Governments and relevant organizations play critical roles in providing food safety information and guiding people toward food choices.

## Figures and Tables

**Table 1:** Demographic characteristics and habits of VF consumption of consumers.

Characteristic		Total (*n* = 456) ^b^	Rural Consumers (*n* = 359) ^b^	Urban Consumers (*n* = 97) ^b^	*p* Value
Age (years)(mean ± SD.) ^a^		40.3 ± 11.8	40.3 ± 11.8	40.3 ± 11.9	0.993
Gender	Male	130 (28.5)	99 (27.6)	31(32.0)	0.447
	Female	326 (71.5)	260 (72.4)	66 (68.0)	
Marital status	Single	163 (35.8)	113 (31.5)	50 (51.5)	0.001 **
	Married	265 (58.1)	224 (62.4)	41 (42.3)	
	Divorced/widowed	28 (6.1)	22 (6.1)	6 (6.2)	
Education	Primary education	19 (4.2)	18 (5.0)	1 (1.0)	0.015 *
	Secondary education	75 (16.4)	66 (18.4)	9 (9.3)	
	Bachelor’s degree or higher	362 (79.4)	275 (76.6)	87 (89.7)	
Monthly income	No income	11 (2.4)	8 (2.2)	3 (3.1)	<0.001 **
	<285 US dollars	69 (15.1)	66 (18.4)	3 (3.1)	
	285–857 US dollars	212 (46.5)	171 (47.6)	41 (42.3)	
	>857 US dollars	164 (36.0)	114 (31.8)	50 (51.5)	
Occupation	Farmers	34 (7.5)	33 (9.2)	1 (1.0)	0.019 *
	Merchants	54 (11.8)	42 (11.7)	12 (12.4)	
	Officers	342 (75.0)	267 (74.4)	75 (77.3)	
	Housewife	10 (2.2)	8 (2.2)	2 (2.1)	
	Student	16 (3.5)	9 (2.5)	7 (7.2)	
Children in the family	Yes	244 (53.5)	205 (57.1)	39 (40.2)	0.004
	No	212 (46.5)	154 (42.9)	58 (59.8)	
Smoking cigarettes	Yes	16 (3.5)	16 (4.5)	0 (0)	0.029
	No	440 (96.5)	343 (95.5)	97 (100)	
Alcohol consumption	Yes	112 (24.6)	87 (24.2)	25 (25.8)	0.791
	No	344 (75.4)	272 (75.8)	72 (74.2)	
Co-morbidity	Yes	99 (21.7)	76 (21.2)	23 (23.7)	0.581
	No	357 (78.3)	283 (78.8)	74 (76.3)	
Frequency of VF purchasing	Always	175 (38.4)	137 (38.2)	38 (39.2)	0.906
	Sometimes	281 (61.6)	222 (61.8)	59 (60.8)	
Source of VF purchasing	Market	348 (76.3)	288 (80.2)	60 (61.8)	<0.001 **
	Supermarket	83 (18.2)	48 (13.4)	35 (36.1)	
	Own plantation	25 (5.5)	23 (6.4)	2 (2.1)	
Type of VF purchasing	Organic	184 (40.4)	132 (36.8)	52 (53.6)	0.025 *
	Pesticide-free ^c^	133 (29.2)	113 (31.5)	20 (20.6)	
	Limit pesticides used	95 (20.8)	78 (21.7)	17 (17.5)	
	Pesticides used	44 (9.6)	36 (10.0)	8 (8.3)	

* *p* value < 0.05; ** *p* value < 0.01; VF, vegetables and fruits; SD, standard deviation. All parameters, except age, are presented as data with *n* (%). ^a^ data analyzed with independent *t*-test; ^b^ data analyzed with chi-square; ^c^ VF were grown and produced without pesticides used.

**Table 2:** Knowledge, attitude, and behavior of consumers to reducing pesticide residues in VF.

Parameters	Rural Consumers		Urban Consumers		*p* Value
	Mean ± SD.	Median (P^25th^, P^75th^)	Mean ± SD.	Median (P^25th^, P^75th^)	
**Knowledge**					
Types of VF products ^a^	3.99 ± 1.09	4 (3, 5)	3.29 ± 1.13	3 (2, 4)	<0.001 **
Purchasing of VF products ^b^	5.03 ± 0.82	5 (5, 6)	4.83 ± 1.09	5 (4, 6)	0.148
Cleaning of VF products ^a^	8.81 ± 2.38	8 (7, 11)	8.38 ± 2.44	8 (7, 10)	0.120
Health effects from pesticides ^b^	1.89 ± 0.41	2 (2, 2)	1.81 ± 0.53	2 (2, 2)	0.126
Total knowledge scores ^b^	19.72 ± 3.15	20 (18, 22)	18.31 ± 3.39	19 (16, 21)	<0.001 **
**Attitude**					
Total attitude scores ^b^	5.00 ± 1.12	5 (5, 6)	4.70 ± 1.32	5 (4, 6)	0.017 *
**Behavior**					
Types of VF products ^a^	22.45 ± 4.34	22 (18, 25)	22.27 ± 4.32	22 (18, 25)	0.716
Purchasing of VF products ^a^	28.94 ± 5.00	29 (25, 32)	28.03 ± 4.74	28 (24, 31)	0.109
Cleaning of VF products ^a^	27.04 ± 5.84	26 (23, 29)	25.09 ± 5.28	24 (22, 28)	0.003 **
Method of cooking ^b^	7.81 ± 1.49	8 (7, 9)	6.98 ± 1.42	7 (6, 7)	<0.001 **
**Total behavior scores** ^a^	86.24 ± 13.77	85 (76, 93)	82.37 ± 12.36	80 (73.5, 90.5)	0.013 *

* *p* value < 0.05; ** *p* value < 0.01; VF, vegetables and fruits; SD, standard deviation; P^25th^, 25th percentile; P^75th^, 75th percentile; ^a^ data analyzed with independent *t*-test; ^b^ data analyzed with Mann–Whitney U test.

**Table 3:** Factors associated with consumers’ behavior in reducing pesticide residues in VF (*n* = 456).

Factors	β	SE.	95%CI	*p* Value
Total knowledge scores	1.15	0.18	0.79, 1.51	<0.001 **
Total attitude scores	1.30	0.49	3.87, 10.40	0.009 **
Co-morbidity	3.21	1.37	0.52, 5.90	0.019 *
Frequency of VF purchasing (sometimes/always)	3.81	1.18	1.49, 6.13	0.001 **
Type of VF purchasing (pesticides used/limit pesticide used/pesticide-free/organic)	1.98	0.57	0.85, 3.11	0.001 **
Price of VF products	−2.23	1.13	−4.45, −0.02	0.048 *
Information from broadcasting tower in community	7.13	1.66	0.32, 2.27	<0.001 **

* *p* value < 0.05; ** *p* value < 0.01; VF, vegetables and fruits; β, beta; SE., standard error; 95%CI, 95% confidence interval.

## Data Availability

Not applicable.

## Supplementary Materials

The following supporting information can be downloaded at: https://www.mdpi.com/article/10.3390/ijerph192013033/s1, Figure S1. Theoretical framework of this study. Figure S2. Districts of Chiang Mai Province. Supplementary S1. Questionnaire form.
