# Supplementary material for: Determinants of Consumers’ Behavior in Reducing Pesticide Residues in Vegetables and Fruits, Northern Thailand
Source: Int J Environ Res Public Health. 2022 Oct 11;19(20):13033. doi: 10.3390/ijerph192013033 (PMC9602482; doi:10.3390/ijerph192013033)
Supplement: Supplementary file 1 [file ijerph-19-13033-s001.zip › ijerph-1944220-supplementary.pdf]

Supplementary S1

Questionnaire Form

Determinants of Consumers' Behavior in Reducing Pesticide Residues in Vegetables and Fruits, Northern Thailand

<u>Part 1: Socio-demographic characteristics:</u>

1.District……………………………., Chiang Mai Province

2.Age……years

3.Gender ☐Male ☐Female

4.Marital status ☐Single ☐Married ☐Divorced/widow

5.Education ☐Primary education ☐Secondary education ☐Bachelor degree or higher

6.Monthly income ☐No income ☐<285 US Dollars ☐285-857 US Dollars ☐>857 US Dollard

7.Occupation ☐Farmers ☐Merchants ☐Officers ☐Housewife ☐Student

8.Children in the family ☐Yes ☐No

9.Smoking status ☐Yes ☐No

10.Alcohol consumption ☐Yes ☐No

11.Co-morbidity ☐ Yes ☐ No

<u>Part 2: Habits of VF consumption.</u>

12.Frequency of vegetables and fruits (VF) purchasing ☐Always ☐Sometimes ☐Never

13.Source of VF purchasing ☐Market ☐Supermarket ☐Own plantation

14.Type of VF purchasing ☐Organic ☐Pesticide-free ☐Limit pesticide used ☐Pesticides used

15.Source of information about pesticides (answer more than 1 choice)

15.1Internet ☐Yes ☐No

15.2TV ☐Yes ☐No

15.3Family or friends ☐Yes ☐No

15.4Academic institutes ☐Yes ☐No

15.5Broadcasting tower in village ☐Yes ☐No

15.6Radio ☐Yes ☐No

16.Considerations for purchasing VF (answer more than 1 choice)

16.1Pesticide-free ☐Yes ☐No

16.2Freshness ☐Yes ☐No

16.3Good for health ☐Yes ☐No

16.4Price ☐Yes ☐ No

16.5Label ☐Yes ☐No

16.6Method of plantation ☐Yes ☐No

16.7Taste ☐Yes ☐No

16.8Packaging ☐Yes ☐No

<u>Part 3: Knowledge in reducing pesticide residues in VF.</u>

17.Knowledge

**Questions**	**Answer**
17.1 Plantation of organic VF can use synthetic hormones for planting	☐Yes ☐No
17.2 Plantation of pesticide-free VF do not used any pesticides for planting	☐Yes ☐No
17.3 Plantation of safe VF do not used any pesticides for planting	☐Yes ☐No
17.4 This symbol means "Pesticide-free VF"	☐Yes ☐No
17.5 This symbol means "Organic VF"	☐Yes ☐No

17.6 This symbol means “Pesticides used VF”	☐Yes ☐No
17.7 You should buy VF which a good appearance and fresh look.	☐Yes ☐No
17.8 You should buy VF which is free of pests and worm bites.	☐Yes ☐No
17.9 You should buy VF which have standard certification.	☐Yes ☐No
17.10 You should buy seasonal VF.	☐Yes ☐No
17.11 You should buy exotic VF.	☐Yes ☐No
17.2 You should buy VF in locally.	☐Yes ☐No
17.13 You should clean VF by using sodium bicarbonate.	☐Yes ☐No
17.14 You should clean VF by using rice vinegar.	☐Yes ☐No
17.15 You should clean VF by rinsing with water 5-10 minutes	☐Yes ☐No
17.16 You should clean VF by peeling the outer parts of VF.	☐Yes ☐No
17.17 You should clean VF by soaking with potassium permanganate.	☐Yes ☐No
17.18 You should clean VF by soaking with salt solution	☐Yes ☐No
17.19 Organic VF is safe for consumers and environment	☐Yes ☐No
17.20 Pesticide-free VF is safe for consumers and environment	☐Yes ☐No
17.21 Exposure to pesticides can increase risk of cancers.	☐Yes ☐No

Part 4: Attitude of pesticide residues in VF

18.Attitude

18.1 Do you worry about the effects of pesticide residues in VF on own health? ☐Yes ☐No
18.2 Do you worry about effects of pesticide residues in VF on your children health? ☐Yes ☐No
18.3 Do you think that purchasing non-pesticide VF is worthwhile? ☐Yes ☐No
18.4 Do you worry about the effects of pesticide residues in VF on environment? ☐Yes ☐No
18.5 Do you need to plant VF for own eating? ☐Yes ☐No
18.6 Do you feel sick because of pesticide residues in VF? ☐Yes ☐No

Part 5: Behavior in reducing pesticide residues in VF

19.Behavior

**Questions**	**Answer**
19.1 You buy organic vegetables.	☐Always ☐Often ☐Sometimes ☐Never
19.2 You buy pesticide-free vegetables.	☐Always ☐Often ☐Sometimes ☐Never
19.3 You buy safe vegetables.	☐Always ☐Often ☐Sometimes ☐Never
19.4 You buy pesticides used vegetables.	☐Always ☐Often ☐Sometimes ☐Never
19.5 You purchase organic fruits.	☐Always ☐Often ☐Sometimes ☐Never
19.6 You purchase pesticide-free fruits.	☐Always ☐Often ☐Sometimes ☐Never
19.7 You buy safe fruits.	☐Always ☐Often ☐Sometimes ☐Never

19.8 You buy pesticides used fruits.	☐Always ☐Often ☐Sometimes ☐Never
19.9 You buy VF which has standard certification.	☐Always ☐Often ☐Sometimes ☐Never
19.10 You buy VF which has a good appearance and fresh look.	☐Always ☐Often ☐Sometimes ☐Never
19.11 You buy VF which is free of pests and worm bites.	☐Always ☐Often ☐Sometimes ☐Never
19.12 You buy seasonal VF.	☐Always ☐Often ☐Sometimes ☐Never
19.13 You buy local VF.	☐Always ☐Often ☐Sometimes ☐Never
19.14 You buy exotic VF.	☐Always ☐Often ☐Sometimes ☐Never
19.15 You buy VF from reliable sources.	☐Always ☐Often ☐Sometimes ☐Never
19.16 You buy VF which has label as "Organic VF"	☐Always ☐Often ☐Sometimes ☐Never
19.17 You buy VF which has label as "Pesticide-free VF"	☐Always ☐Often ☐Sometimes ☐Never
19.18 You buy VF which has label as "Safe pesticide VF"	☐Always ☐Often ☐Sometimes ☐Never
19.19 You buy VF which has label as "pesticides used VF"	☐Always ☐Often ☐Sometimes ☐Never
19.20 You clean VF before eating	☐Always ☐Often ☐Sometimes ☐Never
19.21 You clean VF by soaking with sodium bicarbonate.	☐Always ☐Often ☐Sometimes ☐Never
19.22 You clean VF by soaking with rice vinegar.	☐Always ☐Often ☐Sometimes ☐Never
19.23 You clean VF by soaking with water.	☐Always ☐Often ☐Sometimes ☐Never
19.24 You clean VF by rinsing with water.	☐Always ☐Often ☐Sometimes ☐Never
19.25 You clean VF by peeling the outer part of VF.	☐Always ☐Often ☐Sometimes ☐Never
19.26 You clean VF by boiling method.	☐Always ☐Often ☐Sometimes ☐Never
19.27 You clean VF by soaking with potassium permanganate.	☐Always ☐Often ☐Sometimes ☐Never
19.28 You clean VF by soaking with salt solution.	☐Always ☐Often ☐Sometimes ☐Never
19.29 You clean VF by soaking with commercial VF washing liquid.	☐Always ☐Often ☐Sometimes ☐Never
19.30 You soak VF $\geq$15 minutes	☐Always ☐Often ☐Sometimes ☐Never
19.31 You cook food oneself.	☐Always ☐Often ☐Sometimes ☐Never
19.32 You buy food from market, restaurant, and/or food shop.	☐Always ☐Often ☐Sometimes ☐Never
19.33 You plant VF for own eating in family.	☐Always ☐Often ☐Sometimes ☐Never

---Thank you for your answer---

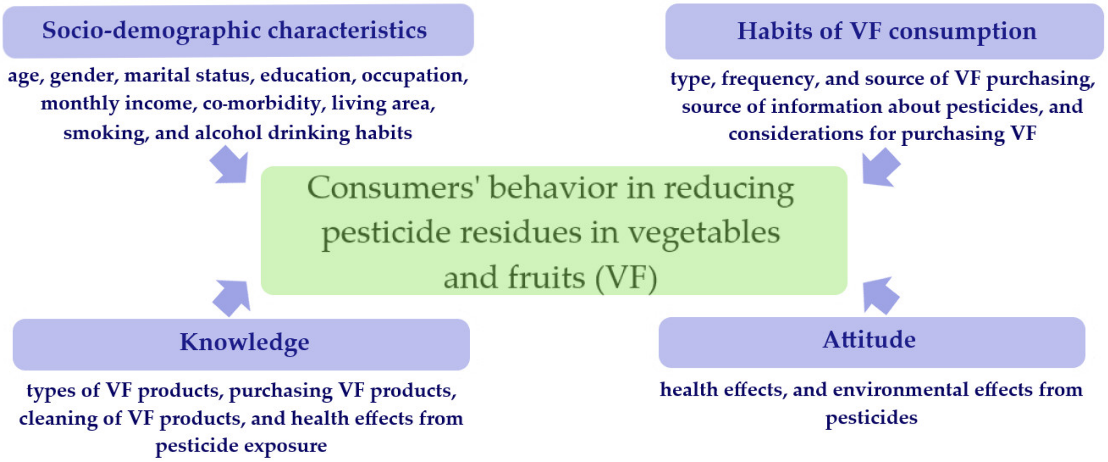

Figure S1. Theoretical framework of this study

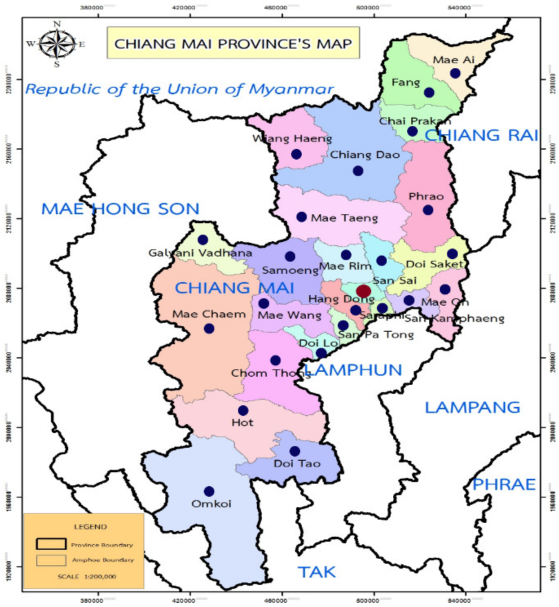

Figure S2. Districts of Chiang Mai Province

- Urban community (Mueang Chiang Mai District)
- Rural community (Mae Ai, Fang, Chai Prakan, Wing Haeng, Chiang Dao, Phroa, Mae Taeng, Doi Saket, San Sai, Mae Rim, Samoeng, Galyani Vadhana, Hang Dong, Mae On, Saraphi, San Kampaeng, San Pa Tong, Mae Wang, Doi Lo, Mae Chaem, Chom Thong, Hot, Doi Tao, and Omkoi Districts)

Source: Chiang Mai Government Center. Available online: http://www.chiangmai.go.th/english/index.php/welcome/information. (accessed on 12 November 2021). [21]
